# Supplementary material for: Methamphetamine Induces Metallothionein 1 Expression and an Inflammatory Phenotype in Primary Human HIV-Infected Macrophages
Source: Int J Mol Sci. 2025 Sep 12;26(18):8875. doi: 10.3390/ijms26188875 (PMC12470063; doi:10.3390/ijms26188875)
Supplement: Supplementary file 1 [file ijms-26-08875-s001.zip › ijms-3683203-supplementary.pdf]

Supplemental Figures:

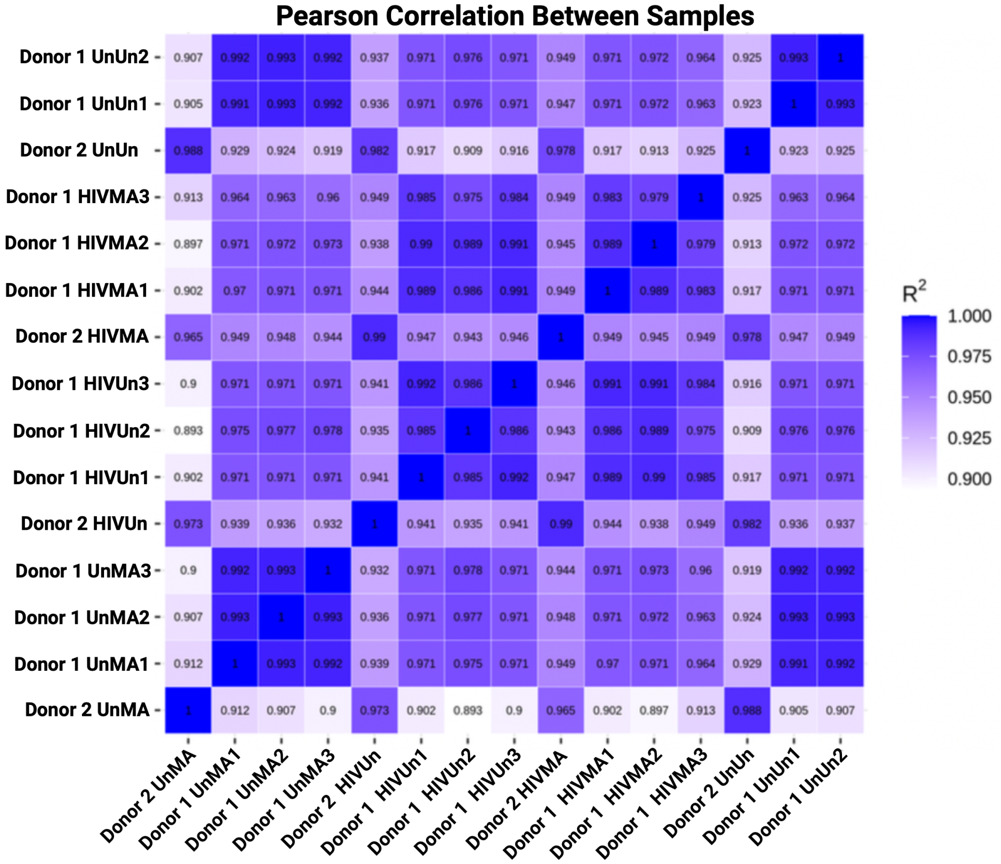

Supplemental Figure S1: <u>RNA Sequencing of Technical Replicates yields highly similar results among replicates</u>
Chart displaying Pearson correlation coefficient values between RNA-seq samples. Donor 1 was cultured on duplicate plates and left uninfected and triplicate plates were infected and all were treated as described. Technical replicates were sequenced to determine if the variability we observe is a technical error of sequencing or biological variation. Conditions indicated on the x and y planes of the graph: UnUn= uninfected untreated, UnMA= uninfected meth treated, HIVUn= HIV-infected untreated, HIVMA= HIV-infected meth treated. Numbers indicate technical replicates. R^2 Values are indicated inside the squares, R^2>0.98 is considered to be highly similar.

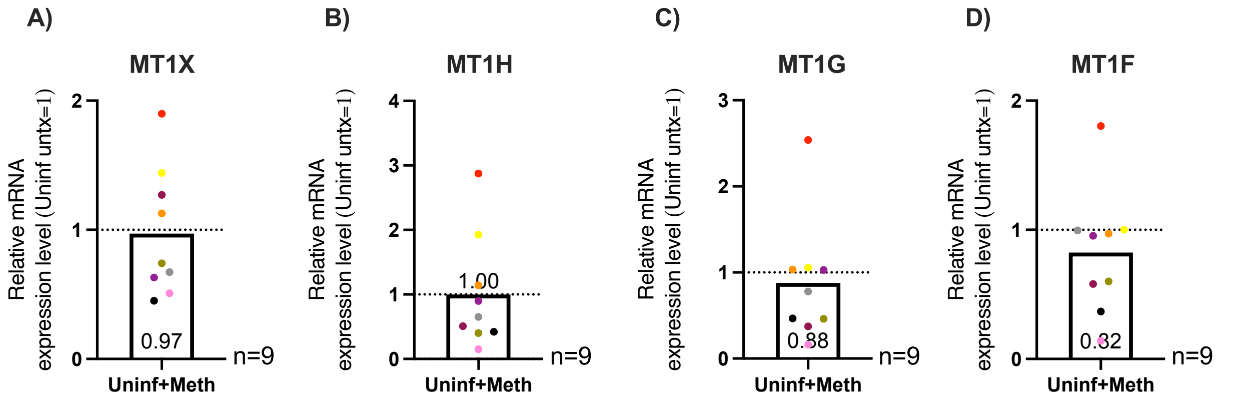

Supplemental Figure S2: Methamphetamine does not change MT1 gene expression in uninfected macrophages

PBMC derived macrophages were left uninfected and treated with meth daily for five days, beginning at the same day post isolation as HIV-infected macrophages. RNA was extracted, cDNA synthesized, and qPCR performed to quantify MT1 gene expression at the end of five days of treatment. Graphs show relative mRNA expression levels of methamphetamine treatment compared to untreated, which is set to one, indicated by the dashed line. Bars represent mean. Colored points correspond to individual donors. “Uninf untx”= uninfected untreated. Significance determined by one sample t-test used for **S2A** (p=0.8647), **S2B** (p=0.9936), and **S2D** (p=0.3030); Wilcoxon signed-rank test used for **S2C** (p=0.4258).

Relative mRNA expression levels- MT1X

	Day 1	Day 2	Day 3	Day 4	Day 5
Donor by color					
Red	N/A	**4.099**	N/A	3.211	1.023
Blue	0.938	**1.239**	0.286	0.41	0.433
Dark purple	1.168	**3.159**	0.908	0.781	0.982
Light green	1.078	0.625	0.581	**2.566**	0.994
Gray	N/A	**1.162**	0.598	0.942	0.458
Orange	1.111	0.741	**1.353**	1.018	0.537
Teal	1.133	**1.703**	1.348	1.107	0.62
Light purple	**4.819**	1.225	2.014	0.644	1.118
Pink	**2.125**	0.527	0.88	0.536	1.15
Dark Green	0.548	1.473	1.02	0.47	**2.83**

Relative mRNA expression levels- MT1H

	Day 1	Day 2	Day 3	Day 4	Day 5
Donor by color					
Red	N/A	0.835	**10.469**	1.09	0.705
Blue	**1.644**	0.596	0.347	0.221	0.535
Dark purple	2.105	**8.491**	0.813	1.063	1.213
Light green	1.048	0.834	0.789	**2.366**	0.816
Gray	N/A	0.986	0.388	**1.289**	0.507
Orange	**1.305**	0.614	0.896	0.981	0.588
Teal	0.978	**1.597**	1.408	0.847	0.63
Light purple	1.409	2.051	0.974	**2.053**	1.387
Pink	1.163	0.371	1.303	0.381	**2.734**
Dark Green	0.561	1.611	**4.868**	0.599	2.851

Relative mRNA expression levels- MT1G

	Day 1	Day 2	Day 3	Day 4	Day 5
Donor by color					
Red	N/A	1.585	**5.405**	1.126	0.75
Blue	**1.941**	1.416	0.321	0.243	0.485
Dark purple	**2.056**	1.118	0.375	0.862	1.378
Light green	0.733	**1.968**	1.624	1.74	1.22
Gray	N/A	1.294	0.408	**1.445**	0.51
Orange	0.602	0.318	0.742	0.661	**1.02**
Teal	1.574	0.874	0.416	0.751	**3.07**
Light purple	0.503	0.359	0.741	0.988	**1.194**
Pink	**1.228**	0.948	1.073	0.789	0.977
Dark Green	**1.566**	0.662	0.872	0.774	1.373

Relative mRNA expression levels- MT1F

	Day 1	Day 2	Day 3	Day 4	Day 5
Donor by color					
Red	N/A	1.608	**1.841**	1.285	N/A
Blue	0.352	**0.847**	0.428	0.407	0.319
Dark purple	**1.696**	1.158	0.664	0.864	1.639
Light green	1.13	0.988	**1.372**	1.312	1.254
Gray	N/A	**1.158**	0.256	0.829	0.696
Orange	0.58	0.444	1.115	0.723	**1.96**
Teal	0.993	1.182	0.941	0.452	**1.641**
Light purple	0.673	1.042	0.971	0.904	**1.2**
Pink	**1.036**	0.939	0.665	0.729	0.826
Dark Green	**2.14**	0.559	0.592	0.809	1.443

Relative mRNA expression levels- MT1E

	Day 1	Day 2	Day 3	Day 4	Day 5
Donor by color					
Red	N/A	1.527	1.861	**2.005**	N/A
Blue	N/A	**1.163**	0.573	0.356	0.387
Dark purple	**2.362**	1.73	0.682	1.305	2.091
Light green	0.86	0.557	0.778	**2.02**	0.944
Gray	N/A	**1.459**	0.393	1.016	0.869
Orange	**1.852**	0.631	0.994	1.168	1.008
Teal	1.019	1.464	0.595	0.753	**2.89**
Light purple	1.263	0.483	**1.644**	0.719	1.083
Pink	**2.368**	1.289	1.775	0.527	1.104
Dark Green	0.75	2.986	0.871	0.526	**3.993**

Supplemental Figure S3: qRT-PCR of daily MT1 gene expression of individual donors
Charts representing daily gene expression values of meth treatment compared to untreated for each donor for each MT1 gene after each day of treatment. Where indicated N/A, there was either not enough cells initially for this treatment timepoint or not enough RNA/cDNA sample. Maximal relative mRNA expression values are shown in colored and bolded font. Font color corresponds to individual donors.

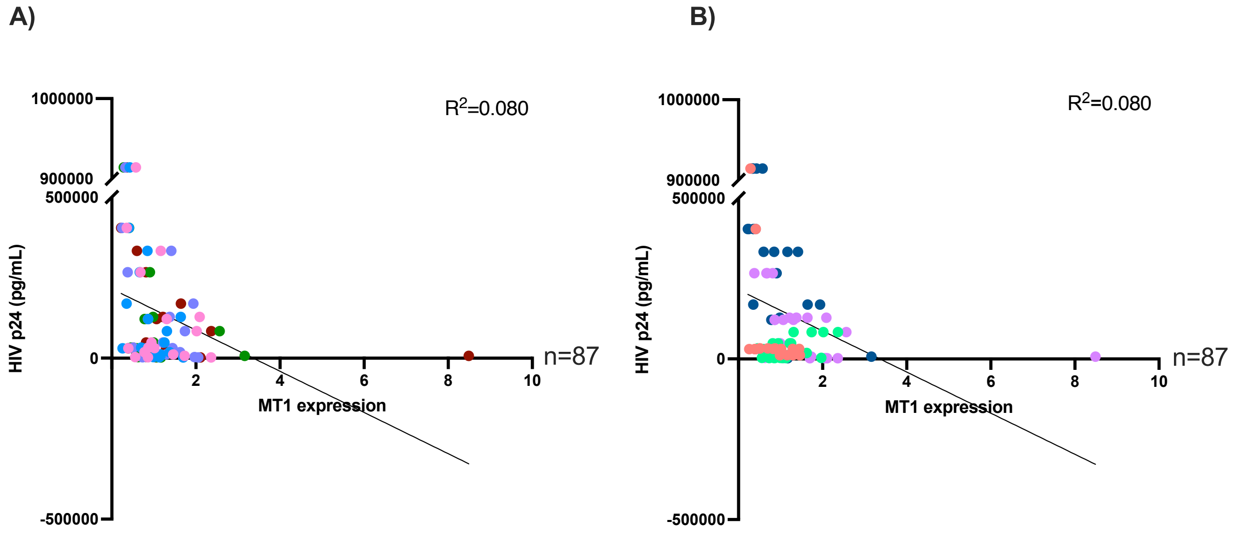

Supplemental Figure S4: There is no correlation between MT1 gene expression and p24 levels
Graphs show HIV p24 measured in supernatants and MT1 gene expression levels. Data from each day of meth treatment for all MT1 genes are represented. In Figure **S4A** dots are colored by MT1 gene, in Figure **S4B**, dots are colored by donor. Linear correlation analysis shows no correlation between p24 levels and each MT1 gene. R^2 values for each individual gene are: MT1X: 0.1029, MT1H: 0.0712, MT1G: 0.211, MT1F: 0.2315, MT1E: 0.1056. Since R^2 values for each gene are below 0.5, there is no correlation between MT1 gene expression level and HIV infection levels.

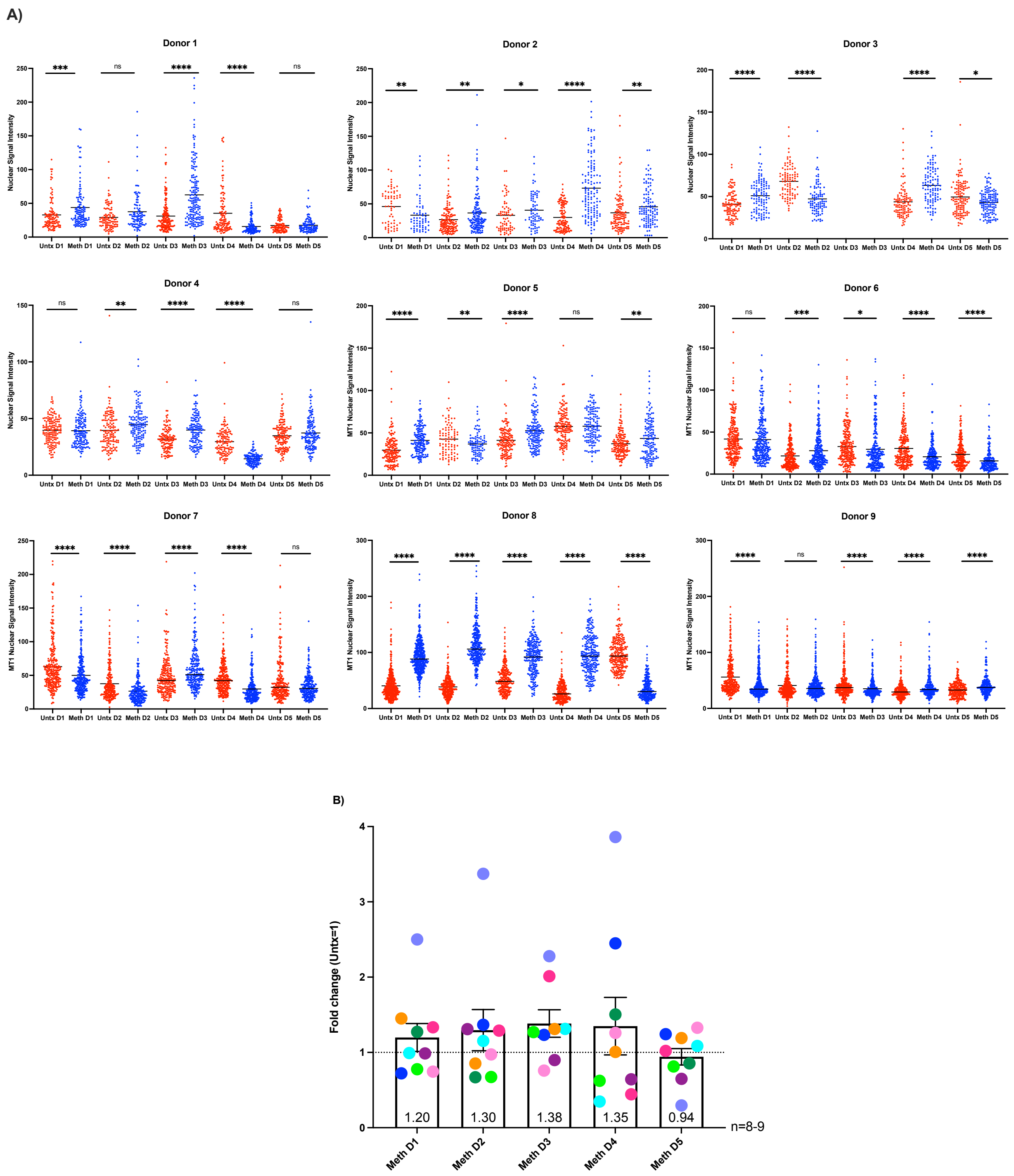

<u>Supplemental Figure S5:</u> <u>MT1 nuclear localization for each donor</u>

S5A) Graphs of daily MT1 signal per nucleus for each donor are shown. Untreated is shown in red and meth treated is shown in blue for each day of treatment. Each point represents a single nucleus's mean fluorescence intensity for MT1 signal. Lines represent mean fluorescence intensity per condition. N=9 independent donors total. "Untx" refers to untreated cells. Donor numbers indicated on top of graphs correspond to the following colors in S5A:

Donor 1= dark pink, Donor 2= royal blue, Donor 3= dark green, Donor 4= light blue, Donor 5= orange, Donor 6= dark purple, Donor 7= light green, Donor 8= light purple, Donor 9= light pink

Significance determined by Wilcoxon matched pairs signed rank test for all donors all paired conditions. *=p>0.05, **=p>0.01, ***=p>0.001, ****=p>0.0001.

S5B) Nuclear MT1 fold change values where HIV untreated=1, as indicated by the dashed line for each day of meth treatment for 8-9 independent donors. Data were generated using the plots in supplemental figure S5A for each donor. Colored points correspond to individual donors. Significance determined by Wilcoxon signed rank test for **D1** (p=0.4961), **D2** (p=0.4961), and **D4** (p=0.8203). One sample t-test was used for **D3** (p=0.073) and **D5** (p=0.6091).

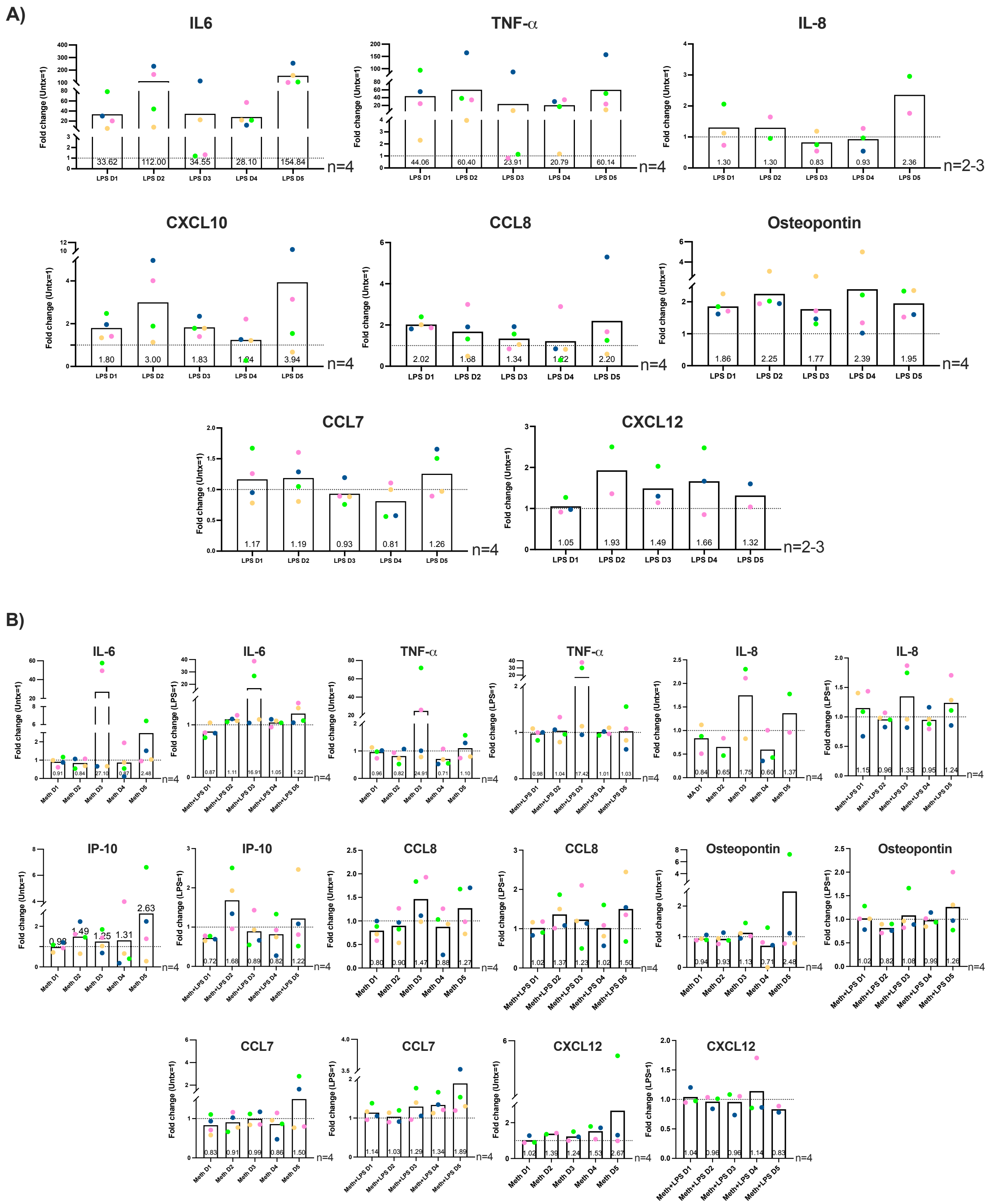

A)
IL6
Fold change (Untx=1)
33.62
112.00
34.55
28.10
154.84
LPS D1
LPS D2
LPS D3
LPS D4
LPS D5
n=4
TNF-α
Fold change (Untx=1)
44.06
60.40
23.91
20.79
60.14
n=4
IL-8
Fold change (Untx=1)
1.30
1.30
0.83
0.93
2.36
n=2-3
CXCL10
Fold change (Untx=1)
1.80
3.00
1.83
1.24
3.94
n=4
CCL8
Fold change (Untx=1)
2.02
1.68
1.34
1.22
2.20
n=4
Osteopontin
Fold change (Untx=1)
1.86
2.25
1.77
2.39
1.95
n=4
CCL7
Fold change (Untx=1)
1.17
1.19
0.93
0.81
1.26
n=4
CXCL12
Fold change (Untx=1)
1.05
1.93
1.49
1.66
1.32
n=2-3
B)
IL-6
Fold change (Untx=1)
Meth D1
Meth D2
Meth D3
Meth D4
Meth D5
n=4
IL-6
Fold change (LPS=1)
Meth+LPS D1
Meth+LPS D2
Meth+LPS D3
Meth+LPS D4
Meth+LPS D5
n=4
TNF-α
Fold change (Untx=1)
0.96
0.82
24.91
0.71
1.10
n=4
TNF-α
Fold change (LPS=1)
0.98
1.04
17.42
1.01
1.03
n=4
IL-8
Fold change (Untx=1)
MA D1
0.84
0.65
1.75
0.60
1.37
n=4
IL-8
Fold change (LPS=1)
1.15
0.96
1.35
0.95
1.24
n=4
IP-10
Fold change (Untx=1)
0.96
1.49
1.25
1.31
2.63
n=4
IP-10
Fold change (LPS=1)
0.72
1.68
0.89
0.82
1.22
n=4
CCL8
Fold change (Untx=1)
0.80
0.90
1.47
0.88
1.27
n=4
CCL8
Fold change (LPS=1)
1.02
1.37
1.23
1.02
1.50
n=4
Osteopontin
Fold change (Untx=1)
0.94
0.93
1.13
0.71
2.48
n=4
Osteopontin
Fold change (LPS=1)
1.02
0.82
1.08
0.99
1.26
n=4
CCL7
Fold change (Untx=1)
0.83
0.91
0.99
0.86
1.50
n=4
CCL7
Fold change (LPS=1)
1.14
1.03
1.29
1.34
1.89
n=4
CXCL12
Fold change (Untx=1)
1.02
1.39
1.24
1.53
2.67
n=4
CXCL12
Fold change (LPS=1)
1.04
0.96
0.96
1.14
0.83
n=4

Supplemental Figure S6: Daily values for inflammatory mediators per donor

S6A) Graphs of daily fold change values for each inflammatory mediator are shown. Graphs show fold change values for LPS treatment compared to untreated set to one for each donor. Colored points correspond to individual donors. "Untx" refers to untreated cells.

S6B) Graphs of daily fold change values for each inflammatory mediator are shown. Graphs show fold change values for meth treatment compared to untreated set to one for every mediator and fold change values for meth+LPS compared to LPS set to one for every mediator. Meth is on the left and meth+LPS is on the right for each mediator. Colored points correspond to individual donors. "Untx" refers to untreated cells.

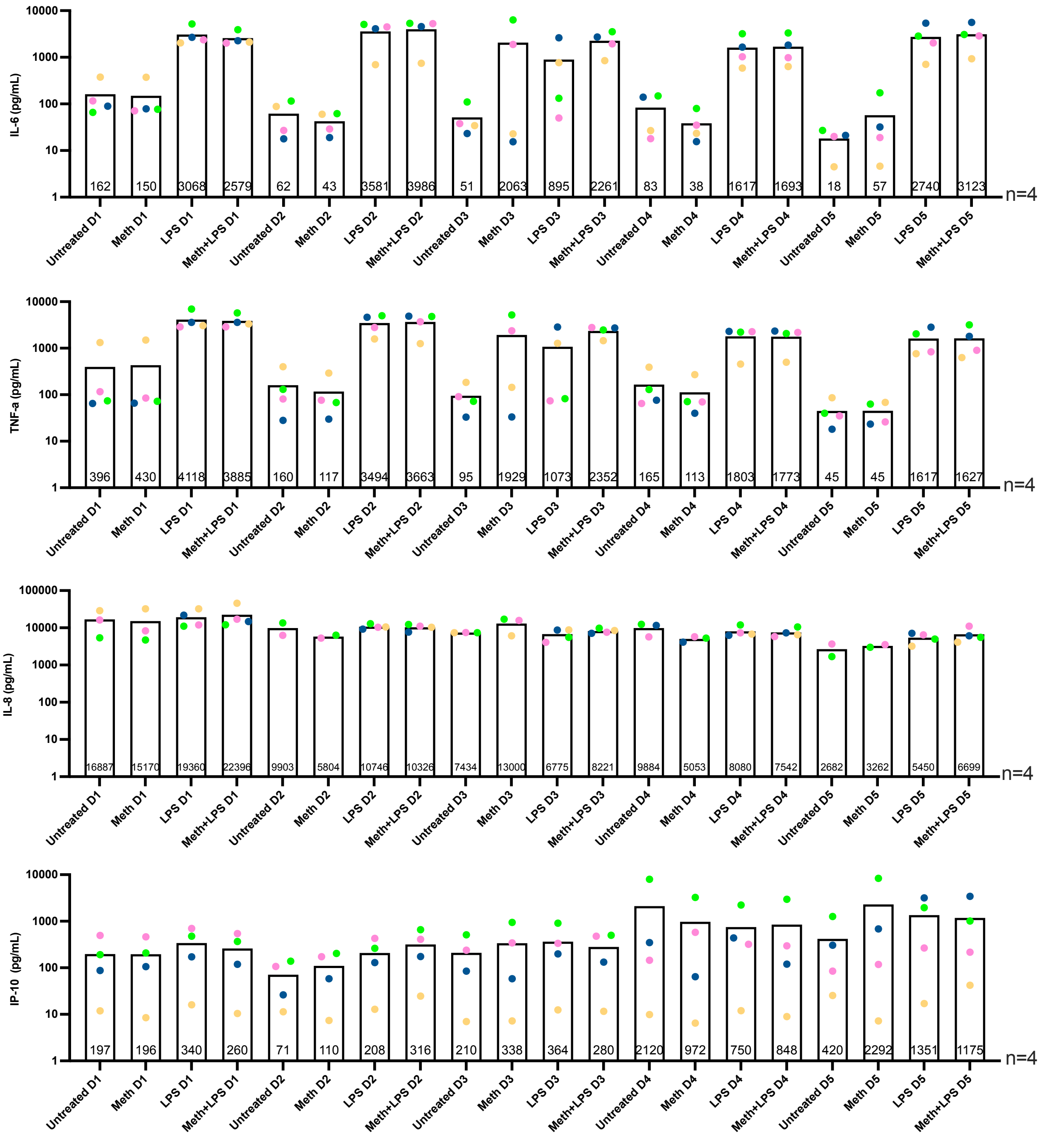
IL-6 (pg/mL)
10000
1000
100
10
1
162 150 3068 2579 62 43 3581 3986 51 2063 895 2261 83 38 1617 1693 18 57 2740 3123
n=4
Untreated D1 Meth D1 LPS D1 Meth+LPS D1 Untreated D2 Meth D2 LPS D2 Meth+LPS D2 Untreated D3 Meth D3 LPS D3 Meth+LPS D3 Untreated D4 Meth D4 LPS D4 Meth+LPS D4 Untreated D5 Meth D5 LPS D5 Meth+LPS D5
TNF-a (pg/mL)
10000
1000
100
10
1
396 430 4118 3885 160 117 3494 3663 95 1929 1073 2352 165 113 1803 1773 45 45 1617 1627
n=4
Untreated D1 Meth D1 LPS D1 Meth+LPS D1 Untreated D2 Meth D2 LPS D2 Meth+LPS D2 Untreated D3 Meth D3 LPS D3 Meth+LPS D3 Untreated D4 Meth D4 LPS D4 Meth+LPS D4 Untreated D5 Meth D5 LPS D5 Meth+LPS D5
IL-8 (pg/mL)
100000
10000
1000
100
10
1
16887 15170 19360 22396 9903 5804 10746 10326 7434 13000 6775 8221 9884 5053 8080 7542 2682 3262 5450 6699
n=4
Untreated D1 Meth D1 LPS D1 Meth+LPS D1 Untreated D2 Meth D2 LPS D2 Meth+LPS D2 Untreated D3 Meth D3 LPS D3 Meth+LPS D3 Untreated D4 Meth D4 LPS D4 Meth+LPS D4 Untreated D5 Meth D5 LPS D5 Meth+LPS D5
IP-10 (pg/mL)
10000
1000
100
10
1
197 196 340 260 71 110 208 316 210 338 364 280 2120 972 750 848 420 2292 1351 1175
n=4
Untreated D1 Meth D1 LPS D1 Meth+LPS D1 Untreated D2 Meth D2 LPS D2 Meth+LPS D2 Untreated D3 Meth D3 LPS D3 Meth+LPS D3 Untreated D4 Meth D4 LPS D4 Meth+LPS D4 Untreated D5 Meth D5 LPS D5 Meth+LPS D5

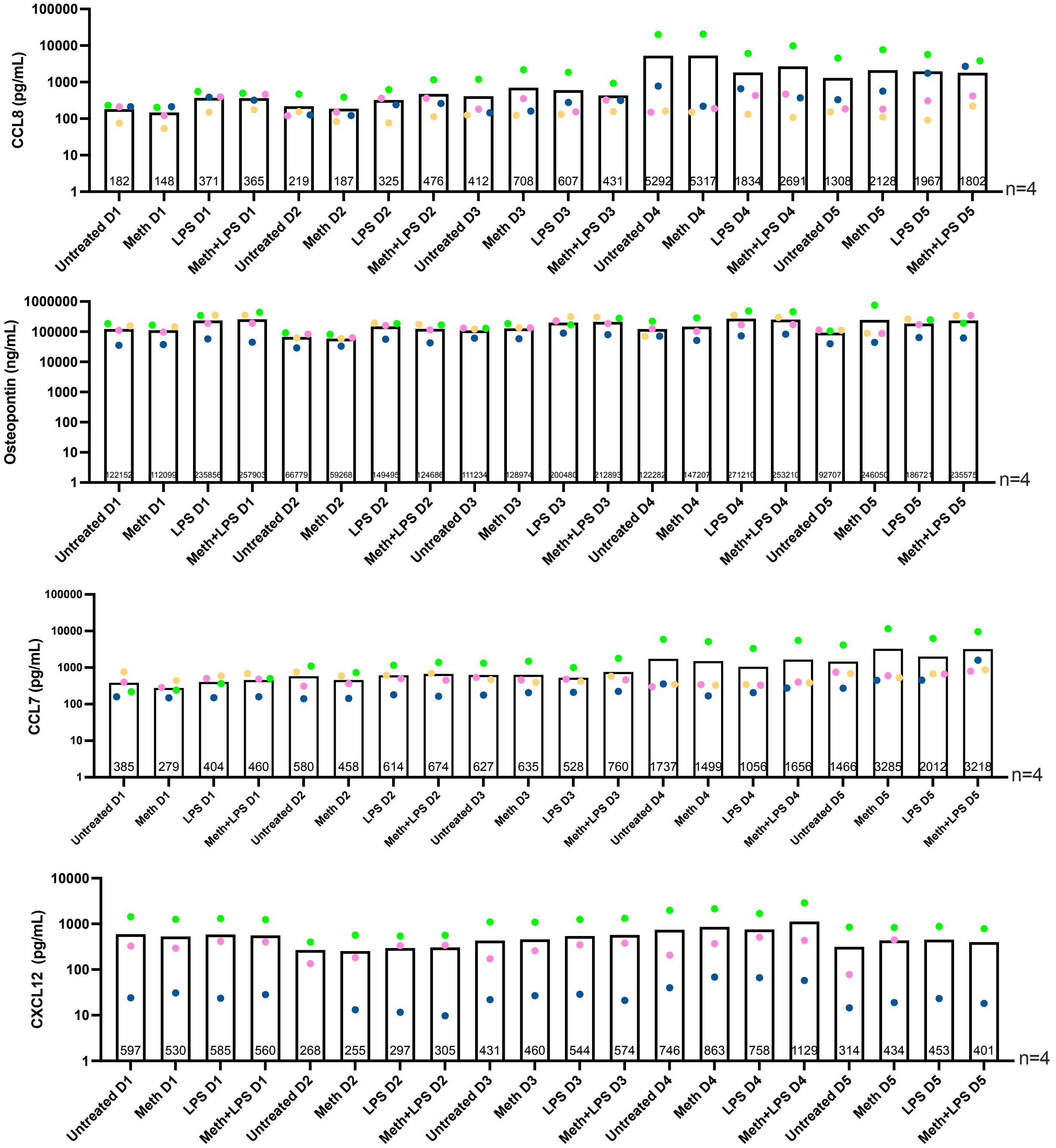

Supplemental Figure S7: Values for inflammatory mediators

Graphs show concentrations of mediators reported in pg/mL. Sample dilutions were assessed to yield values on the linear portion of the curve. Standard curve R^2 values were 0.98 or greater. Values were used to generate fold change values where untreated is set to one. Colored points correspond to individual donors.
